# The effect of opium addiction on short-term postoperative outcomes after coronary artery bypass graft surgery: A prospective observational cohort study

**DOI:** 10.37796/2211-8039.1042

**Published:** 2020-12-01

**Authors:** Mohammad Reza Habibi, Abbas Alipour, Aria Soleimani, Farshad Hasanzadeh Kiabi, Ali Habibi, Amir Emami Zeydi

**Affiliations:** a Department of Anesthesiology, Faculty of Medicine, Mazandaran University of Medical Sciences, Sari, Iran; b Department of Community Medicine, Faculty of Medicine, Mazandaran University of Medical Sciences, Sari, Iran; c Faculty of Medicine, Mazandaran University of Medical Sciences, Sari, Iran; d Department of Medical-Surgical Nursing, Nasibeh School of Nursing and Midwifery, Mazandaran University of Medical Sciences, Sari, Iran

**Keywords:** Coronary artery bypass, Postoperative complications, Intraoperative complications, Opium dependence

## Abstract

**Introduction:**

Opium addiction has been recently suggested as a potential risk factor for the occurrence of perioperative complications in patients undergoing coronary artery bypass graft (CABG) surgery. The aim of this study was to evaluate whether opium addiction can potentially affect patients' short-term postoperative outcomes after CABG surgery.

**Material and methods:**

In a prospective observational cohort study, all consecutive patients who were scheduled for first-time isolated elective on pump CABG surgery were screened during the study period for opium addiction. The study was carried out between September 2015 and November 2016 at Mazandaran Heart Center, Sari, Iran. A total number of 228 patients [110 opium addicted (OA) and 118 non-addicted (NA)] were screened and included. All patients were evaluate, in terms of short-term postoperative outcomes, until hospital discharge or death.

**Results:**

In the OA patients, the mean amount of estimated postoperative bleeding was significantly more than NA patients (535 ± 304.75 ml vs. 463.56 ± 209.77; P = 0.04). Mean ventilation time were significantly longer in the OA patients than in the NA (9.9 days vs. 8.66 days, P = 0.02). The mean duration of postoperative hospital stay was two days longer in the OA (10.83 days vs. 8.34 days, P < 0.001). Also, the mean use of packed cell during surgery and incidence of postoperative atrial fibrillation were higher in the OA patients than NA (P = 0.005).

**Conclusion:**

The results of our study provide strong evidence that the opium addiction should be considered as a risk factors for developing perioperative complications, including higher mean postoperative bleeding, need for intraoperative packed red blood cell transfusion, ventilation time and length of hospital stay, in patients undergoing CABG surgery.

## 1. Introduction

In recent years the prevalence of coronary artery disease (CAD) is increasing, worldwide [1–4]. Coronary artery bypass (CABG) surgery is a treatment approaches in patients with CAD [5–7]. It is estimated that approximately 500 thousand CABG surgeries performed annually in the United States [8–10]. CABG surgery is considered as a high-risk surgery due to the nature of the surgery and the potential for perioperative complications [11–15]. Despite the all recent technological advances and improved surgical techniques, perioperative complications remain one of the most important causes of morbidity in patients undergoing CABG surgery [12–16]. Bleeding, dysrhythmias particularly atrial fibrillation, perioperative myocardial infarction (MI), renal dysfunction and surgical site infection are among the more common complications following CABG surgery [16,17]. These complications can result in significant patients' mortality, morbidity, longer intensive care unit (ICU) and hospital stay and consequently impose additional cost on both health system and patient [12,16]. It has been shown that approximately 10% of the healthcare costs and resource utilization in patients undergoing CABG surgery is associated with the occurrence of perioperative complications [16]. Hence, recognizing patients with high risk of perioperative complications and managing them with proper prophylactic interventions will significantly reduce the associated morbidity, mortality and costs [18–21].

Opium addiction has been recently suggested as a potential risk factor for the occurrence of perioperative complications in patients undergoing CABG surgery [22]. Addiction is a social and health problem in many countries, including Iran [22–25]. It is believed that Iran experiences the 2nd most severe addiction to opioids in the world [25,26]. Although it has been shown that opioids addiction is associated with increased risk of all-cause mortality, including cardiovascular diseases and cancer [24], one reason for the high prevalence of opium use among Iranian population is the misconception among some people that opioid use could prevent or ameliorate diabetes, hypertension and cardiac diseases [27]. It has been shown that the prevalence of opioid addiction in Iranian patients undergoing CABG surgery is high, ranging from approximately 9% to 16% [28–32]. Therefore, it is important to pay special attention to the opioid addiction and its' potential consequences in patients undergoing CABG [22].

Relatively few studies evaluate the relationship between opioid addiction and the occurrence of perioperative complications after cardiac surgery. However, with conflicting results, it is difficult to understand clearly the relationship between opioid addition and perioperative complications in patients undergoing CABG surgery [27,29–33]. Therefore this study aimed to investigate whether opium addiction can potentially affect patients' short-term postoperative outcomes after CABG surgery.

## 2. Material and methods

After obtaining approval from the institutional Ethics Committee and informed written consent from participants, all adult patients (age, 35-75 years), with American Society of Anesthesiologists (ASA) physical status class I and II, who were scheduled for first-time isolated elective on pump CABG surgery were included in this prospective observational cohort study. The study was conducted at Mazandaran Heart Center, Sari, Iran. Patients with previous history of cardiac surgery, heart failure with ejection fraction (EF) < 30%, renal failure (serum creatinine > 1.5 mg/dL on two consecutive tests), need for more than 4 grafts, and combined CABG with cardiac valve surgery were excluded from the study.

All consecutive patients were assessed during the study period for opium addiction and were assigned to one of the two groups [opium addicted (OA) and non-addicted (NA)]. Diagnosis of opium addiction was made according to the Diagnostic and Statistical Manual of Mental Disorders (DSM-IV TR) criteria; if at least 3 of 7 behavioral, cognitive and physiological effects are meet by the patients in the past 12 months. Non-addicted patients never used opium in their lives before surgery. Patients’ demographic and clinical characteristics including sex, age, history of diabetes mellitus (DM), body mass index (BMI), history of hypertension (HTN), myocardial infarction (MI), cigarette smoking, and preoperative EF were recorded. In patients with opium addiction, duration and the route of opium consumption was also recorded. Moreover, prior to and on days 1 and 2 after the surgery (at 8:00 a.m. each day), from all patients the blood samples were gotten to measure the levels of hemoglobin (Hb), blood urea nitrogen (BUN), hematocrit (HCT) and creatinine (Cr).

In both two groups the protocols for anesthesia, cardiopulmonary bypass (CPB), and surgery were same. For adequate anticoagulation state during surgery, the initial dose of heparin (300 IU/kg) was given to achieve a minimum activated clotting time (ACT) of 480 seconds. After CPB weaning, heparin was reversed by full dose of protamine chloride to achieved pre-programed target of ACT (<120 seconds). Also, for all patients mild hypothermia (32°C) was employed during CPB.

After surgery, all patients transferred to the cardiac surgery intensive care unit (ICU). Intra and postoperative information including duration of surgery, time of cardiopulmonary bypass, cross-clamp time, mechanical ventilation duration and the grafts number were evaluated and recorded for all patients. Also, information on perioperative transfusion of platelets, packed red blood cells (PRBC) and fresh frozen plasma (FFP), incidences of re-operation for the control of bleeding, needing to intra aortic balloon pump (IABP) and needing to inotropic drugs, and also length of hospital satay were recorded for all patients by an ICU nurse. All patients without significant postoperative bleeding (during the first 6 hour) received heparin infusion (1000 IU/h) for 24 hour. For postoperative pain control during ICU stay, all patients were connected to patient-controlled intravenous analgesia with morphine (10 mg)-paracetamol (Apotel®- 4 gr). All patients in two groups were monitored carefully and continuously during ICU and after being moved to the coronary care unit (CCU). If ICU nurse practitioners visually detect any suspicious rhythms on monitoring, a 12-lead electrocardiogram (ECG) was recorded and then the ECG was assessed by two independent cardiologists blinded to the groups. In postoperative period, occurrence of any dysrhythmia that represents the characteristics of atrial fibrillation and last at least 30 seconds on a rhythm strip or 12-lead ECG were considered as postoperative atrial fibrillation (POAF). All patients were evaluate until hospital discharge or death. For prevention of withdrawal syndrome post-operatively, all OA patients received their total daily doses of opium orally or via a nasogastric tube during hospitalization.

### 2.1. Statistical analysis

Shapiro-Wilk was used to evaluate whether data were normally distributed. Patients' baseline demographic and clinical characteristics for two groups (opium addicted; OA and non-addicted group; NA) were tabularized as percentages, median (interquartile range) or mean ± SD. Also, chi-square or Fisher-exact test and student t-test or Mann-Whitney *U* test were used for categorical data in two groups. Intention-to-treat analysis was used for evaluation postoperative complications. Score of those parameters were analyzed using repeated measurement analysis of variance (ANOVA) test between two groups. P-value of less than 0.05 was considered statistically significant.

## 3. Results

Among 252 screened patients throughout the period of study, 11 patients declined to participate in the study and 13 patients were not to be eligible to include in the study. Finally, 228 patients were enrolled into this study (110 OA and 118 NA). The OA included 35 females (31.8%), and the mean age was 60.15 ± 9.14 years. The NA included 45 females (38.1%), and the mean age was 60.3 ± 9.32 years. Mean of BMI for the OA patients was 26.25 ± 3.95 kg/m2 and for NA patients was 25.82 ± 4.07 kg/m2. No statistically significant differences were observed between two groups in terms of patients' demographic and clinical characteristics (Table 1).

No significant differences were seen in terms of mean preoperative ejection fraction (EF), hemoglobin, hematocrit, creatinine, and albumin levels between two groups (Table 2).

### 3.1. Intraoperative and postoperative outcomes

In the OA patients, the mean of postoperative bleeding volume was more than NA patients (535 ± 80.75 ml vs. 463.56 ± 91.77; P = 0.04). Mean duration of surgery was similar between two group (3.97 ± 1 hour vs.3.94 ± 0.7; P = 0.83). Mean ventilation time were significantly longer in the OA than in the NA (9.9 days vs. 8.66 days, P = 0.02). In the OA patients the mean length of postoperative hospital stay was longer compared to NA patients (10.83 days vs. 8.34 days, P < 0.001). The mean use of packed cell during surgery was higher in the OA than NA (P = 0.005). OA patients had significantly more POAF than the NA patients (P = 0.012). Occurrence of other complications in OA patients was higher than NA ones, but the difference was not statistically significant (P > 0.05) (see Table 3).

Mean pre-and post-operation of EF, Hb, HCT, Cr, and BUN levels of each group has been shown in Table 4. After matching the likely effective variable (DM), the differences of Hb became significant statistically (*p* = 0.01) and the difference of other parameters were not statistically significant (*p* > 0.05). There was a statistically main effect for time (P < 0.01), indicating that regardless of the groups, the mean baseline Hb, and HCT levels was higher than their mean postoperative values and the mean of postoperative values of Cr, and BUN was higher than the baseline values. No patients in both groups needed IABP or re-operation.

## 4. Discussion

The results of our study revealed that opium addiction is associated with significantly higher mean postoperative bleeding, need for intra-operative packed red blood cell transfusion, ventilation time and hospital stay length in patients undergoing CABG surgery. Opium consumption is considerably higher among cardiac patients than the general population in Iran [34]. Additionally, it has been revealed that using opium is about twice as common among Iranian cardiac surgery patients compared to Western countries. Additionally, postoperative cardiac, CNS and respiratory complications are higher in opium dependents and abusers compared to non-substance users [30]. In a study by Ezadi-Mood et al. has been shown that the prevalence postoperative delirium after CABG surgery is higher among opium addicted patients compared to non-addicted patients [35]. The results of another study indicate that opium abuser patients who underwent CABG surgery, compared to non-opium users, had significantly higher mechanical ventilation time after surgery. Also, opium abusers had higher DPB, MAP and HR in the first and second postoperative days, compared to non-user patients [36]. Additionally, opium addiction has been suggested as a predictor of POAF after cardiac surgery; so that patients with opium use had higher rate of POAF after CABG surgery [29,37]. In another study it was found that opium addiction is a risk factor for occurrence of post MI dysrhythmias [38]. A 10 years prospective cohort study shows that opium use in patients is associated with higher mortality rate after CABG surgery [39]. Nemati et al. in their study showed that opium addiction is associated with higher bleeding in postoperative period after CABG surgery [22]. Sadeghian et al. revealed that patients with opium addiction had longer resource utilization during hospitalization following CABG surgery [32]. Also, another study revealed that opium addicted patients undergoing CABG surgery were more likely to readmitted to the hospital, due to cardiac causes, during the 6 month after surgery [31]. Despite the potential negative impact of opium addiction in patients undergoing CABG surgery, some studies suggest protective effects of opium abuse as a protective factors in these patients. In a study by Amini et al. has shown that patients with opium abuse after CABG had significantly less frequent of acute kidney injury compared to non-opium abusers [40]. Also, Maghsoudi et al. reported that the length of inotropic agents in CABG perioperative period was significantly lower in opium addicted compared to non-opium dependent patients [33]. Further studies are required to investigate the potential protective effects of opium in patients undergoing CABG surgery. There are a few limitations in this study. The short term follow-up and comparatively small sample size and of the patients in this study limited it's only indicative value and therefore further studies with larger sample size and longer follow up for assessing the longer outcomes are warranted.

## 5. Conclusion

The results of our study provide strong evidence that the opium addiction should be considered as a risk factors for developing perioperative complications, including higher mean postoperative bleeding, need for intraoperative packed red blood cell transfusion, ventilation time and length of hospital stay after CABG surgery.

## Figures and Tables

**Table 1:** Demographic and comorbidities state according to opium addiction.

Variables	Group		P value
	Opium addicted (N = 110)	Non-addicted (N = 118)	
Age, year, mean ± SD	60.15 ± 9.14	60.3 ± 9.32	0.91
Sex, F/M ratio	35.75	45.73	0.33
BMI, kg/m2, mean ± SD	26.25 ± 3.95	25.82 ± 4.07	0.42
DM	37 (33.61)	52 (44.12)	0.14
HTN	57 (52.83)	57 (48.37)	0.51
Cigarette smoking	73 (66.42)	69 (58.55)	0.27
Number of grafts, median (interquartile range)	3 (3-4)	3 (3-4)	0.63

F/M: female/male; BMI: Body mass index; DM: Diabetes mellitus; HTN: Hypertension.

**Table 2:** Preoperative ejection fraction (EF), hemoglobin, hematocrit, and creatinine levels according to opium use.

Variables	Group		P value
	Opium addicted (N = 110)	Non-addicted (N = 118)	
EF	49.44 ± 7.16	50.92 ± 6.83	0.11
Hb	12.96 ± 1.45	12.67 ± 1.31	0.11
HCT	38.38 ± 4.37	37.79 ± 3.85	0.43
Cr	1.05 ± 0.22	1.07 ± 0.24	0.47
BUN	17.25 ± 4.92	17.76 ± 5.57	0.28

EF: Ejection Fraction; Hb: Hemoglobin; HCT: Hematocrit; Cr: Creatinine; BUN: Blood Urea Nitrogen.

**Table 3:** The perioperative outcomes in OA and NA patients.

Variables	Group		P value
	Opium addicted (N = 110)	Non-addicted (N = 110)	
Duration of surgery, hour	3.97 ± 16	3.94 ± 0.78	0.83
CPB time, min	66.55 ± 21.71	65.76 ± 18.74	0.76
Cross-clamp time, min	44.23 ± 15.11	41.11 ± 13.89	0.11
Ventilation time, hour	9.94 ± 4.43	8.66 ± 3.27	**0.02**
Need to Inotropic drugs	6 (5.52)	3 (2.54)	0.33
Length of hospital stay, day	10.83 ± 6.67	8.34 ± 2.58	<**0.001**
POAF	10 (9.13)	2 (1.76)	**0.01**
Other dysrhythmia	1 (0.91)	0 (0)	0.49
Packed cell use during surgery	0.94 ± 0.84	0.64 ± 0.69	**0.005**
Packed cell use after surgery	0.95 ± 1.16	0.69 ± 0.86	0.13
FFP use in surgery	0.04 ± 0.38	0 ± 0	0.13
FFP use after surgery	0.15 ± 0.64	0.06 ± 0.41	0.48
Platelet use in surgery	0.43 ± 1.39	0.16 ± 0.87	0.32
Platelet use after surgery	1.27 ± 2.46	0.83 ± 1.86	0.13

CPB: Cardiopulmonary bypass; POAF: Postoperative atrial fibrillation; FFP: Fresh frozen plasma.

**Table 4:** Pre-and post-operative EF, Hb, HCT, Cr, and BUN levels of each study group.

Variables		Time			P value	
		Before surgery	One day after surgery	Two day after surgery	Time trend	Time * group interaction
EF	Opium addicted	49.44 ± 7.1	48.22 ± 6.4	——————	0.85	0.6
	Non-addicted	50.92 ± 6.8	50.39 ± 6.27	——————		
Hb	Opium addicted	12.96 ± 1.45	9.5 ± 1	8.43 ± 1.22	<**0.001**	**0.014**
	Non-addicted	12.67 ± 1.31	9.5 ± 1.02	8.74 ± 0.94		
HCT	Opium addicted	38.38 ± 4.37	28.8 ± 2.8	26.01 ± 3.61	<**0.001**	0.12
	Non-addicted	37.79 ± 3.8	28.77 ± 3	26.75 ± 2.71		
Cr	Opium addicted	1.05 ± 0.22	1.14 ± 0.23	1.17 ± 0.29	**0.08**	0.13
	Non-addicted	1.07 ± 0.2	1.11 ± 0.21	1.17 ± 0.26		
BUN	Opium addicted	17.25 ± 4.92	20.45 ± 5.93	25.01 ± 9.45	<**0.001**	0.24
	Non-addicted	17.76 ± 5.57	19.41 ± 6.21	24.16 ± 8.74		

EF: Ejection Fraction; Hb: Hemoglobin; HCT: Hematocrit; Cr: Creatinine; BUN: Blood Urea Nitrogen.
